# Metabolomic alterations in cord blood improve the prediction of childhood-onset neurodevelopmental disorders

**DOI:** 10.1038/s41398-026-04208-x

**Published:** 2026-06-25

**Authors:** Polina Girchenko, Marius Lahti-Pulkkinen, Chenyao Ni, Jari Lahti, Li Tian, Aino Airikka, Eero Kajantie, Katri Räikkönen

**Affiliations:** 1 Clinical Medicine Research Unit, MRC Oulu, University of Oulu and Oulu University Hospital, Oulu, Finland; 2 Department of Psychology, Faculty of Medicine, University of Helsinki, Helsinki, Finland; 3 Finnish Institute for Health and Welfare, Helsinki, Finland; 4 Centre for Cardiovascular Science, Queen’s Medical Research Institute, University of Edinburgh, Edinburgh, United Kingdom; 5 Folkhälsan Research Center, Helsinki, Finland; 6 Department of Clinical and Molecular Medicine, Norwegian University of Science and Technology, Trondheim, Norway; 7 Department of Obstetrics and Gynecology, Helsinki University Hospital, Helsinki, Finland

**Keywords:** Predictive markers, Diagnostic markers

## Abstract

We aimed to identify cord blood metabolic measures associated with childhood-onset neurodevelopmental disorders (NDDs) and to examine whether they improve prediction beyond known early-life risk factors. In a prospective study of 858 children followed to a median age of 14.1 years (IQR 13.7–14.8), cord blood levels of 110 metabolic measures were assessed at birth and NDD diagnoses obtained from nationwide register. We identified 12 cord blood metabolic measures together explaining 5.0% of variance in any NDD, seven related to HDL synthesis, concentration and composition, one to concentration of lipoprotein particles, two to fatty acids and two to amino acids; their levels were 0.26–0.36(95%CI: −0.55, −0.06) standard deviations lower in the children who were later diagnosed with any NDD compared to the children with no mental disorders (p-value for overall model including all 12 metabolic measures <0.0001). They were also associated with early-life risk factors of NDDs, and improved the prediction of any NDD over a model that included only sociodemographic, prenatal and birth-related early-life risk factors(AUC = 0.74,95% CI 0.69–0.79; R2 = 16.6%; p < 0.0001 vs. AUC 0.71,95% CI, 0.66–0.76; R2 = 12.2%; p < 0.0001; Likelihood Ratio Test [LRT] p = 0.04). The improvement was more modest when the model additionally included maternal polygenic risk scores for ADHD and ASD (AUC = 0.75,95% CI 0.70–0.80; R2 = 18.3%; p < 0.0001 vs. AUC 0.72,95% CI, 0.67–0.78; R2 = 13.9%; p < 0.0001; LRT p = 0.08). Cord blood metabolic measures predicted NDDs diagnosed from birth through adolescence and improved prediction beyond early-life risk factors, with smaller incremental value when maternal PRSs for ADHD and ASD were included.

## Introduction

Neurodevelopmental disorders (NDDs), including Attention-Deficit Hyperactivity Disorder (ADHD), Autism Spectrum Disorder (ASD), disorders of intellectual development, and psychological developmental, speech and language, learning, motor coordination, and stereotyped movement disorders [1] are highly prevalent complicating the lives of 15–20% of children [2]. The peak age at diagnosis for NDDs is 5.5 years, with nearly all (95.8%) cases diagnosed by 25 years [3]. NDDs are comorbid with each other and with other mental disorders that may manifest later in life, such as mood disorders and schizophrenia [4], thereby multiplying their lifelong burden. As current approaches to timely diagnosis and treatment have significant limitations, it is important to identify biomarkers indicating risk of NDDs as early in life as possible. This need is particularly relevant within the Developmental Origins of Health and Disease (DOHaD) context, as studies suggest that NDDs may originate in prenatal life [5–14]. During this critical period, exposure to environmental adversities may negatively affect fetal brain development and thereby increase risk for NDDs [15].

Analysis of fetal cord blood metabolome may offer a valuable opportunity to identify such early life biomarkers. Cord blood contains a combination of metabolites transported form the maternal circulation through the placenta to the fetus, as well as metabolites synthesized and metabolized by both the placenta and the fetus [16, 17]. It thereby represents the health status of a newborn baby [18–21]. This unique composition makes cord blood metabolomics an especially powerful tool for capturing the early-life exposures and biological processes that may contribute to the development of NDDs.

Previous research on the associations between the cord blood metabolome and NDDs has produced inconsistent results. We are aware of five studies that have specifically explored the cord blood metabolome in relation to NDD diagnoses [17, 22–25]. The Boston Birth Cohort study assessed a targeted panel of eight cord blood metabolic measures related to branched chain amino acids (AA), tryptophan and other selected metabolites. The study reported that higher levels of leucine, isoleucine, valine, and their composite score derived through factor analysis, were associated with an increased risk of ADHD in the sample of 626 children followed from birth until ages 5 to 19 years [24]. Another study using participants from the same cohort (N = 966) reported that higher levels of 5-methoxytryptophol were associated with a lower risk of ADHD and ASD, whereas higher levels of tryptophan, 5-hydroxytryptophan, and N-acetyltryptophan were associated with a higher risk of ADHD [23]. In the Markers of Autism Risk in Babies, Learning Early Signs study, which measured a targeted panel of 42 cord blood metabolites related to AA, fluid balance, ketone bodies and glycolysis in 124 cord blood samples, higher levels of 3-Hydroxybutyrate, a ketone body, were associated with higher risk of non-typical neurodevelopment, but not ASD in children assessed at age 3 years [17]. The Norwegian Autism Birth Cohort study, measured a targeted and untargeted panel of 1204 cord blood metabolites in 418 children. The study reported that the associations between cord blood metabolic measures and risk of ASD were partially sex-specific. In both girls and boys the risk of ASD was associated with alterations in primary metabolites, biogenic amines and complex lipids, whereas in boys also with oxylipins [22]. The Irish BASELINE study measured an untargeted panel of cord blood metabolites from 22 children diagnosed with ASD before the age of 5 and 44 neurotypical controls. The study identified dysregulation in metabolites associated with glycolysis, oxygen transport, and steroid metabolism in children with ASD [25]. Previous studies are, however, limited by their primary focus on ADHD and ASD, and with the exception for the Norwegian and Irish studies, by the use of restricted metabolite panels and analytic methods that do not fully capture the complex interrelationships within metabolomic data. Furthermore, no studies to date have explored how prenatal environmental adversities and associated birth outcomes may contribute to cord blood metabolic changes linked to NDDs. Understanding these associations could provide valuable insights into the biological mechanisms linking prenatal adversities with NDDs.

Therefore, the aim of this study was to identify cord blood metabolic measures associated with a broader range of NDDs than examined in the previous studies. Our primary outcome was any NDD diagnosis in a cohort of 858 children followed from birth to 12.4–16.8 years of age [3]. We used a targeted panel of 110 cord blood metabolic measures covering core metabolic pathways, including lipoproteins, AA, fatty acids (FA), glycolysis-related metabolites, ketone bodies, fluid balance and inflammation. This panel has been widely applied in epidemiological studies and 37 of its metabolic measures have been clinically validated. We also evaluated whether the identified metabolic measures were associated with and improved the prediction of NDDs beyond established early-life risk factors, including sociodemographic, prenatal and birth-related factors, and maternal NDDs operationalized through maternal polygenic liabilities for ADHD and ASD. We also studied whether the identified metabolic measures were associated with subclinical symptoms of NDDs.

## Methods and materials

The cohort study was approved by the by the ethics committees of the Helsinki and Uusimaa Hospital District, and register data were linked with the permission from the register authority. All participants and guardian(s) of minors provided written informed consent. The Strengthening the Reporting of Observational Studies in Epidemiology (STROBE) reporting guideline was used.

### Study population

The Prediction and Prevention of Pre-eclampsia and Intrauterine Growth Restriction (PREDO) study enrolled 1079 pregnant mothers and their singleton children born alive between 2006 and 2010. Women were recruited based on their risk factor status for preeclampsia and intrauterine growth restriction from 10 study hospitals in Southern and Eastern Finland at their first ultrasound screening at 12–14 gestational weeks [26]. Of the mothers, 979 provided fasting blood samples for DNA extraction. This sample was enriched by 72 women who donated their placentas for research purposes but whose risk factor status for preeclampsia and intrauterine growth restriction at the recruitment was unknown At birth, cord blood samples were collected from 923 newborns. Of these children, 921 (99.8%) had diagnostic data on mental and behavioral disorders; 63 children had mental and behavioral disorders other than NDDs and were excluded from the analytic sample. Children included in the analytic sample (N = 858) (Table 1) had a higher gestational age at birth [39.8 (SD 1.6) vs. 39.0 (SD 2.8) gestational weeks; p < 0.0001] and a higher birth weight z-score [0.00 (SD 1.01) vs. −0.16 (SD 1.18) SD;p = 0.03] than the excluded children. However, there was no evidence of systematic differences with regard to any other early-life risk factors (p > 0.05). Maternal polygenic liabilities for ADHD and ASD were studied in a subsample comprising 737 mother-child pairs. The children have been followed from birth through Finnish nationwide registers up to December 2022, at which point they were between 13.1 and 16.8 years old. In addition, clinical follow-ups were conducted when the children were 2.0–5.7 and 7.0–12.1 years old.

**Table 1:** Early-life risk factors of neurodevelopmental disorders in the children with neurodevelopmental disorders and in children with no mental or behavioral disorders in a follow-up from birth to 12.4–16.8 years.

Early-life risk factors	No mental or behavioral disorders (N = 738)	Any NDD (N = 120)	ADHD (N = 51)	Speech and language disorders (N = 43)	Scholastic skills disorders (N = 32)	ASD (N = 24)	Motor development disorders (N = 20)
	M (SD) or No. (%)	M (SD) or No. (%)	M (SD) or No. (%)	M (SD) or No. (%)	M (SD) or No. (%)	M (SD) or No. (%)	M (SD) or No. (%)
**Sociodemographic risk factors**							
Maternal age, years	33.0 (5.5)	33.0 (6.0)	31.4 (6.1)*	33.5 (5.9)	31.0 (7.0)*	33.6 (5.5)	33.0 (6.5)
Maternal education, No. (%)		***	*	*	***		
Primary or secondary education	303 (42.0%)	69 (59.0%)	29 (60.4%)	26 (61.9%)	23 (76.7%)	9 (31.1%)	14 (73.7%)
University degree	418 (58.0%)	48 (41.0%)	19 (39.6%)	16 (38.1%)	7 (23.3)	14 (60.9%)	5 (26.3%)
Data not available	17 (2.3%)	3 (2.5%)	3 (5.8%)	1 (2.3%)	1 (3.1%)	1 (4.1%)	1 (5.0%)
Maternal smoking and/or alcohol use during pregnancy, No. (%)							
No	613 (83.1%)	95 (79.2%)	40 (78.4%)	38 (88.4%)	27 (84.8%)	19 (79.2%)	16 (80.0%)
Yes	125 (16.9%)	25 (20.8%)	11 (21.6%)	5 (11.6%)	5 (15.6%)	5 (20.8%)	4 (20.0%)
**Prenatal risk factors**							
Maternal pre-pregnancy body mass index, kg/m^2^	26.7 (6.2)	29.4 (6.5)***	30.1 (7.0)***	29.6 (6.1)**	29.5 (7.3)*	27.3 (4.2)	31.4 (5.6)***
Maternal diabetes disorders during pregnancy, No. (%)				*			**
No	527 (71.4%)	76 (63.3%)	33 (64.7%)	26 (60.5%)	21 (65.6%)	18 (75.0%)	10 (50.0%)
Yes	211 (28.6%)	44 (36.7%)	18 (35.3%)	17 (39.5%)	11 (34.4%)	6 (25.0%)	10 (50.0%)
Maternal hypertensive disorders during pregnancy, No. (%)				**			
No	509 (69.0%)	77 (64.2%)	33 (64.7%)	21 (48.8%)	23 (71.9%)	16 (66.7%)	11 (55.0%)
Yes	229 (31.0%)	43 (35.8%)	18 (35.3%)	22 (51.2%)	9 (28.1%)	8 (33.3%)	9 (45.0%)
Maternal antenatal depression, No. (%)		***	***	***	**	***	
No	700 (94.9%)	101 (84.2%)	41 (80.4%)	34 (79.1%)	26 (81.3%)	16 (66.7%)	17 (85.0%)
Yes	38 (5.2%)	19 (15.8%)	10 (20.8%)	9 (20.9%)	6 (18.7%)	8 (33.3%)	3 (15.0%)
**Polygenic liabilities for ADHD and ASD**							
Polygenic risk score for ADHD of the mother, SD	−0.05 (1.00)	0.21 (0.96)*	0.28 (0.91)*	0.32 (1.03)*	0.26 (0.94)	0.31 (0.82)	0.25 (1.06)
Polygenic risk score for ASD of the mother, SD	−0.02 (0.99)	0.00 (1.02)	0.14 (1.11)	−0.05 (0.98)	−0.20 (1.18)	0.35 (1.10)	−0.09 (1.27)
Data not available	105 (14.4%)	16 (13.3%)	7 (13.7%)	5 (11.6%)	4 (12.5%)	4 (16.7%)	1 (5.0%)
**Birth outcomes**							
Child sex, No. (%)		***	***	***	**	***	**
Boy	364 (49.3%)	86 (71.7%)	40 (78.4%)	36 (83.7%)	25 (78.1%)	21 (87.5%)	16 (80.0%)
Girl	374 (50.7%)	34 (28.3%)	11 (21.6%)	7 (16.3%)	7 (21.9%)	3 (12.5%)	4 (20.0%)
Child gestational age at birth, weeks	39.8 (1.6)	39.8 (1.8)	39.7 (1.5)	39.8 (1.9)	39.8 (1.5)	39.9 (2.2)	39.3 (2.4)
Birth weight z-score, standard deviation units	−0.04 (1.03)	0.19 (1.0)*	0.23 (1.13)	0.24 (1.13)*	0.34 (0.91)*	0.04 (1.26)	0.21 (1.23)

There is no missing data unless indicated otherwise.

Comparison of characteristics between children with any NDD or specific NDDs and children without mental or behavioral disorder diagnoses: *p < 0.05 **p < 0.01 ***p < 0.001.

### Outcomes

NDD diagnoses, coded according to International Statistical Classification of Diseases and Related Health Problems, 10th Revision (ICD-10), were derived from the Care Register for Healthcare (HILMO). The validity of HILMO register diagnoses of mental disorders have been shown to be good [27, 28]. Any NDD diagnoses vs. no diagnoses of mental or behavioral disorders was the primary outcome. Median (Interquartile range) age at first NDD diagnosis was 14.3 (13.6–15.0) years. As secondary outcomes we focused on the most prevalent specific NDD diagnoses: ADHD [n = 51, (5.9%)], speech and language disorders [n = 43, (5.0%)], scholastic skills disorders [n = 32, (3.7%)], ASD [n = 24, (2.8%)], and motor development disorder [n = 20, (2.3%)] (Table 1, eTable 1, Supplement 1).

Subclinical symptoms of NDDs were assessed using mother-reported developmental milestones from the Ages and Stages Questionnaires (ASQ) [29, 30], and ASD and ADHD symptoms from the Autism Spectrum Screening Questionnaire (ASSQ) [31] and the ADHD Rating Scale [32] (eTable 2, Supplement 1).

### Cord blood metabolomic measures

After the child was born, midwives collected fetal cord blood from the umbilical vein, from which plasma was separated and stored at −80 °C until analysis. A high-throughput proton nuclear magnetic resonance (NMR) metabolomics platform quantified 110 metabolic measures using the Nightingale Health Quantification Library 2020 [33] (Nightingale Health Ltd, Helsinki, Finland). Details of the experimentation and applications of the NMR metabolomics platform have been described previously [34]. In brief, the thawed samples (260 μL) were carefully mixed with sodium phosphate buffer (260 μL) and moved to NMR tubes. The setup is a combination of Bruker AVANCE III 500 MHz (a selective inverse room temperature probe head) and Bruker AVANCE III HD 600 MHz spectrometers (a cryogenically cooled triple resonance probe head, CryoProbe Prodigy TCI), both with the SampleJet robotic sample changer. The lipid extraction procedure was done manually (Integra Biosciences VIAFLO 96 channel electronic pipette) based on multiple extraction steps containing saturated sodium chloride solution, methanol, dichloromethane, and deuterochloroform and data were collected in full automation with the 600 MHz instrument. Computers that controlled the spectrometers do the Fourier transformations to NMR spectra and automated phasing. A centralized server performs various automated spectral processing steps, including overall signal check for missing/extra peaks, background control, baseline removal and spectral area-specific signal alignments and the spectral information was compared to 2 quality control samples. Metabolic measures below level of detection (LOD) were replaced with a value of 0.9 multiplied by the minimum value of that measurement [35]. All metabolic measures were log-transformed and standardized to the mean of 0 and standard deviation (SD) of 1. All values above and below 5 standard deviations (SDs) from the mean were considered outliers and recoded as missing values [36]. Missing values were not imputed and were treated as missing in all analyses. The information on the metabolic measures, mean (SD), percentage of samples below limit of detection are presented in eTable 3 in Supplement 1. ETable 4 additionally presents the raw values of the cord blood metabolic measures that were associated with NDDs, shown separately for children with any NDD and for children with no mental or behavioral disorders.

### Early-life risk factors for NDDs

#### Sociodemographic risk factors

We derived maternal age (years) from the Medical Birth Register (MBR) [37], education level (secondary or lower vs tertiary) was self-reported in early pregnancy, smoking and/or alcohol use during pregnancy (yes vs no) combined data on smoking during pregnancy derived from MBR and self-reported data on alcohol consumption in early pregnancy [13, 36].

#### Prenatal risk factors

Maternal pre-pregnancy obesity (body mass index [BMI] ≥ 30 kg/m^2^) was extracted from the MBR and maternal diabetes (International Statistical Classification of Diseases and Health-Related Problems 10th Revision [ICD-10] codes: O24.4 for gestational diabetes mellitus (GDM); E10 for type 1 diabetes; E11 for type 2 diabetes) and hypertensive disorders during pregnancy (O13 for gestational hypertension; O10. I10 for chronic hypertension; O11. O14. O15 for preeclampsia) were extracted from the MBR. and/or the Care Register for Healthcare (HILMO). The diagnoses of hypertensive disorders were further verified by an expert jury comprising two medical doctors and a research nurse with expertise in obstetrics and gynecology. History of depression diagnoses until childbirth was derived from HILMO (F32-F33. F341 since 1996; ICD-9 codes 2961. 2968 A. and 3004 A from 1987 to1995). In the PREDO study, starting from 12–13 gestational weeks, mothers also completed the Center for Epidemiological Studies Depression Scale (CES-D) biweekly until 38–39 gestational weeks or delivery [26]. We averaged available CES-D scores during pregnancy and used a cutoff of 20 to define clinically relevant depression [38]. We combined diagnostic and symptom data into one dichotomous variable indicating antenatal depression (depression diagnoses or CES-D ≥ 20 vs. no diagnoses and CES-D < 20) [5].

#### Birth outcomes

Data on the child’s sex, gestational age and weight at birth were also available from MBR. Birth weight z-score was calculated using Finnish national growth standards [39].

#### Maternal polygenic liabilities for ADHD and ASD

Genotyping was performed on the IlluminaGlobal Screening array (Illumina Inc, San Diego, CA) at the Erasmus MC, The Netherlands and imputed with IMPUTE 2.3.2 and Eagle v2.3 against Finnish-specific SISu v2 reference panel (GRCh37) comprising 2690 high-coverage whole-genome and 5093 high-coverage whole-exome sequences, which introduces less false polymorphisms than using global reference panels [40]. Before imputation, variants with call rate <0.95, MAF> 0.35, minor allele count <19 or HWE p < 1 × 10 − 6 were excluded. Samples were excluded based on call rate <0.95, heterozygosity F < 0.1, sex mismatch, and relatedness. Population outliers were excluded based on visual inspection. There were 15,544,584 variants after imputation. PPRSs were calculated based on SNP weights provided by the largest available genome-wide association study (GWAS) summary statistics for ADHD [41] and ASD [42] using LDpred2-auto [43] and PLINK (v1.9b.6) [44].

### Statistical analysis

We first conducted exploratory analyses to identify a combination of cord blood metabolic measures collectively associated with any NDD using elastic net logistic regression. We used 10-fold cross-validation because it efficiently uses limited data by allowing all observations to contribute to both training and validation, thus reducing bias and variance in performance estimates and minimizing dependence on any single random split. Ten-fold cross-validation has been shown to offer a good balance between bias and variance compared with other resampling methods [45]. A 10-fold cross-validation was performed at the alpha levels 0.1–0.9 to ensure the optimal balance between L1 and L2 regularization. The penalty parameter value yielding the smallest prediction error was used to achieve the best predictive performance. To evaluate the robustness of the metabolic measures selected by the elastic net models, we performed a bootstrap-based feature stability analysis. We generated 100 bootstrap samples of equal size to the original dataset (sampled with replacement). In each bootstrap sample, we refit the full elastic net logistic regression pipeline using the same tuning parameters as in the primary analysis. Feature stability was quantified as the proportion of bootstrap samples in which each metabolite was selected and directionality of the effect.

All subsequent analyses were confirmatory. To assess the effect sizes of metabolic measures selected by the elastic net regression, while accounting for their high intercorrelation, we applied bivariate linear mixed model (BLMM) [46, 47]. With BLMM, we also studied whether the metabolic measures selected by the elastic net regression differentiated children with the most prevalent specific NDDs, and whether they were associated with subclinical symptoms of NDDs. Similar to the analyses of any NDDs, analyses using specific NDDs as outcomes, the comparison group comprised children without any mental or behavioral disorder diagnoses. The BLMM models were also used to examine associations between the selected metabolic measures and early-life risk factors. Early-life risk factors were also included as covariates in the BLMM models for child NDDs diagnoses. Because maternal PRSs for ADHD and ASD were available only for a subset of mothers, which limits statistical power, we present models adjusted for the PRSs as additional analyses. Metabolite-specific BLMM p-values are post-selection nominal p-values and should be interpreted cautiously. The overall pattern of findings is captured by the BLMM p-value for the full model. Thus, emphasis should be placed on the overall pattern rather than on per-metabolite p-values as confirmatory evidence.

We then evaluate the predictive performance of the two logistic regression models, the first model including sociodemographic and prenatal risk factors and birth outcomes and the second one additionally the cord blood metabolic measures identified by the elastic net regression. Predictive performance of the models was estimated based on the total amount of variance explained in any NDDs by Nagelkerke Pseudo R^2^ and area under the receiver operating characteristic curve (AUC). To compare the fit of the two logistic regression models we applied likelihood ratio test (LRT). We reran the analyses by adding maternal PRSs for ADHD and ASD into the models. Elastic net regression was performed using Stata statistical software version 18 (StataCorp),. All other statistical analyses were performed using R 4.5.2. and SAS 9.4 (SAS Institute Inc., Cary, NC, USA). The computer code and scripts used to generate the results in this study are available from the corresponding author upon reasonable request.

## Results

Of the original sample, cord blood samples were available for 923 newborns. In a follow-up of the children from birth to 12.4–16.8 years, 921 had data available on whether they had or had not been diagnosed with mental or behavioral disorders (ICD-10: F00-F99). Of these children, 120 (13.1%) met the criteria of at least one NDD. The comparison group comprised 738 (80.1%) children who had never received any diagnoses of mental or behavioral disorders. Of the NDDs, the most prevalent were ADHD (N = 51, 5.9%), speech and language disorders (n = 43, 5.0%), scholastic skills disorders (n = 32, 3.7%), ASD (n = 24, 2.8%), and motor development disorders (n = 20, 2.3%) (eTable 1 in Supplement 1). Of the 923 children with cord blood data, neurodevelopmental milestones were available for 460 children at the age of 2.0–5.6 years and subclinical symptoms of ASD and ADHD were available for 415 and 420 (eTable 2 in Supplement 1). Table 1 shows the characteristics of the children with any and the most prevalent NDDs and children with no mental and behavioral disorders. Characteristics of the children included in the sample with data available on maternal PRSs for ADHD and ASD are in eTable 5 in Supplement 1.

### Metabolic measures associated with NDDs

**Fig. 1:**
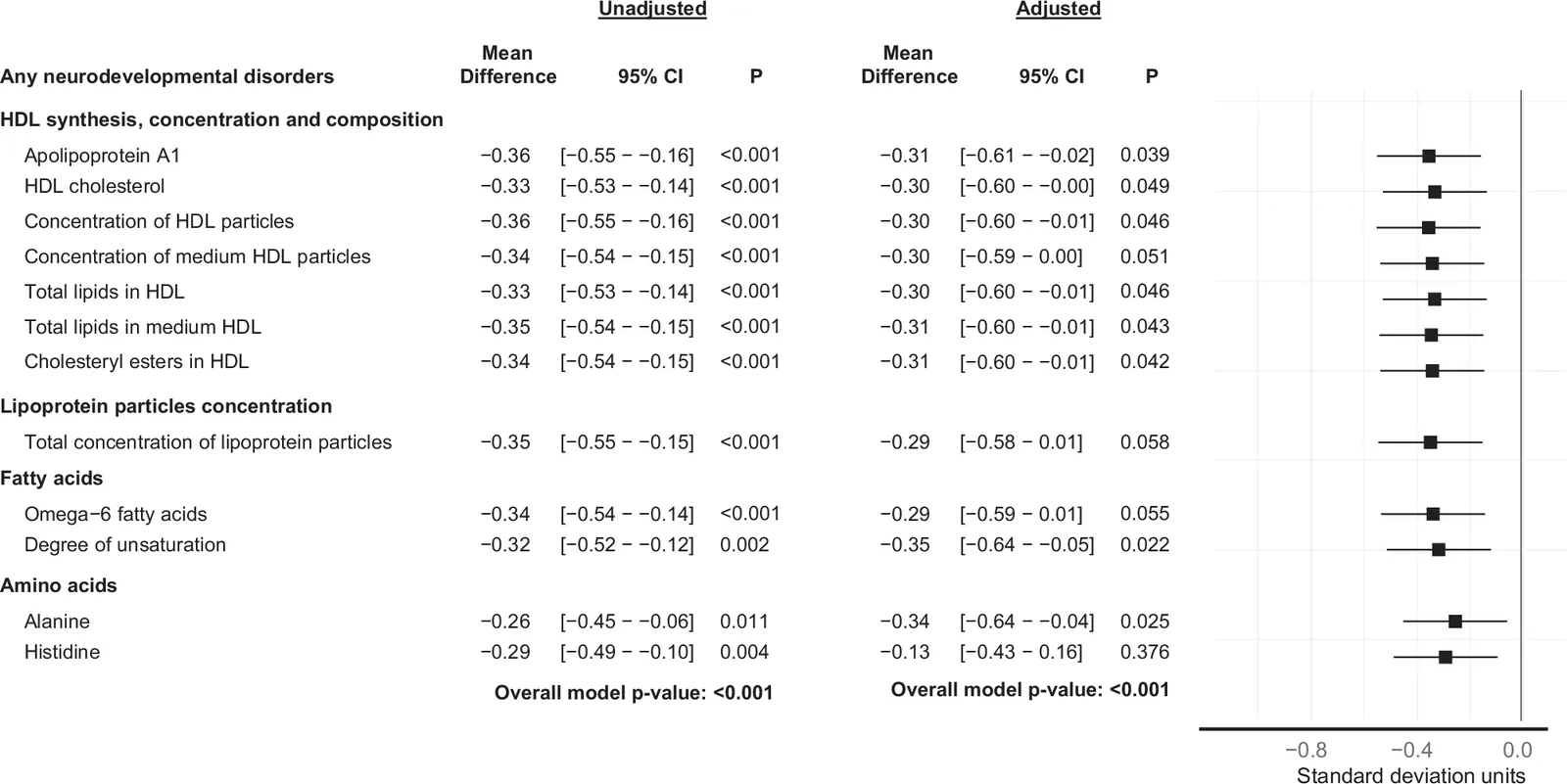
Unadjusted and adjusted mean differences in the 12 cord blood metabolic measures between children with any neurodevelopmental disorders and those with no mental disorders. Adjusted models included early-life risk factors for neurodevelopmental disorders: sociodemographic risk factors (maternal age at delivery, education, smoking and alcohol use during pregnancy), prenatal risk factors (maternal pre-pregnancy body mass index, diabetes and hypertensive disorders during pregnancy, and antenatal depression) and birth outcomes (child sex, gestational age at birth and birth weight z-score). The forest plot displays unadjusted mean differences (squares) and 95% confidence intervals (error bars).

**Fig. 2:**
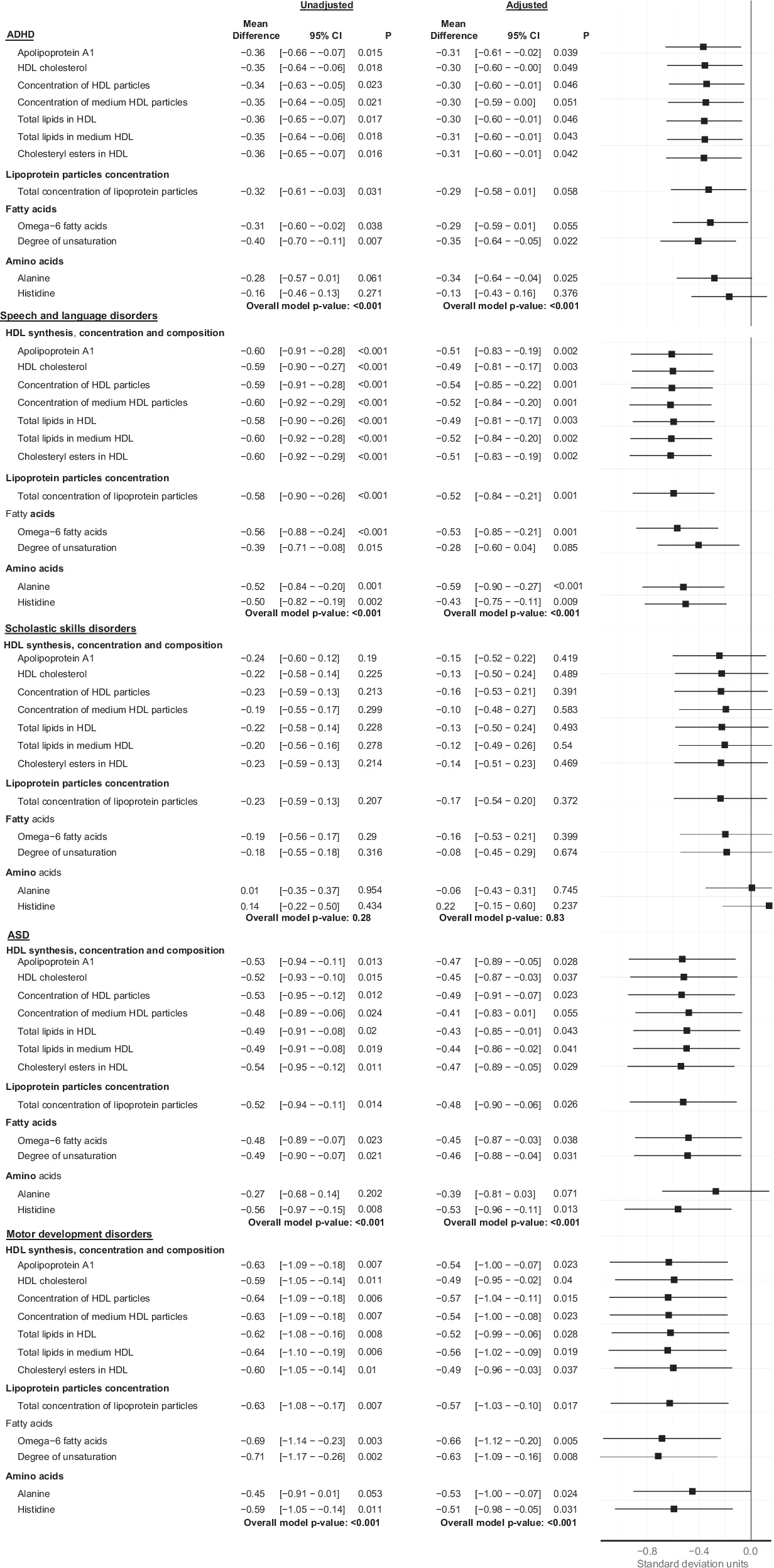
Unadjusted and adjusted mean differences in the 12 cord blood metabolic measures between children with ADHD, speech and language disorders, motor development disorders, and ASD and children with no mental disorders. Adjusted models included early-life risk factors for neurodevelopmental disorders: sociodemographic risk factors (maternal age at delivery, education, smoking and/or alcohol use during pregnancy), prenatal risk factors (maternal pre-pregnancy body mass index, diabetes and hypertensive disorders during pregnancy, and antenatal depression) and birth outcomes (child sex, gestational age at birth and birth weight z score).The forest plots display unadjusted mean differences (squares) and 95% Confidence Intervals (error bars).

Elastic net regression identified 12 cord blood metabolic measures collectively associated with any NDDs. The 12 cord blood metabolic measures explained 5.0% of the variance in any NDDs, Seven of these measures were related to HDL synthesis, concentration and composition (apolipoprotein A1, HDL cholesterol, concentration of HDL particles, concentration of medium HDL particles, total lipids in HDL, total lipids in medium HDL, cholesteryl esters in HDL). The measures also included lipoprotein particles concentration (total concentration of lipoprotein particles), fatty acids (FA) (degree of unsaturation, omega-6 FA) and amino acids (AA) (alanine and histidine). In bootstrap-based feature stability analysis several metabolic measures demonstrated high stability, being selected in ≥80% of the bootstrap samples. The most stable features were apolipoprotein A1 (selection frequency 90%), HDL cholesterol (84%), HDL cholesteryl esters (84%), degree of unsaturation (82%), histidine (82%), and alanine (80%). Other metabolic measures exhibited moderate stability (selection frequencies 68–79%) appearing in the majority of bootstrap resamples. For all 12 metabolic measures, the directionality of the effect in bootstrap-selected features aligned consistently with the direction observed in the primary analysis. All 12 selected cord blood metabolic measures were highly correlated (r > 0.31, p < 0.0001) (eFig. 2 in Supplement 1). Figure 1 shows that the levels of the 12 selected metabolites were 0.26–0.36 SDs lower in the children who later developed any NDD in comparison with the children with no mental or behavioral disorders (p-value for overall model<0.0001). The models including the 12 cord blood metabolic measures also differentiated the children who later developed ADHD, speech and language disorders, ASD and motor development disorders from the children with no mental disorders (p-values for overall models <0.0001) (Fig. 2). The results did not change when adjusted for the early-life risk factors (p-values for overall models <0.0001) (Figs. 1, 2). Additional adjustment of the model testing associations with any NDDs for maternal PRSs for ADHD and ASD did not change the results (eTable 6 in Supplement 1). The overall pattern of associations between the 12 metabolic measures and both developmental milestones and ASD was consistent with the pattern observed for diagnostic outcomes (eFig. 3 in Supplement 1).

### Associations between early-life risk factors of NDDs and the cord blood metabolic measures associated with any NDDs

**Fig. 3:**
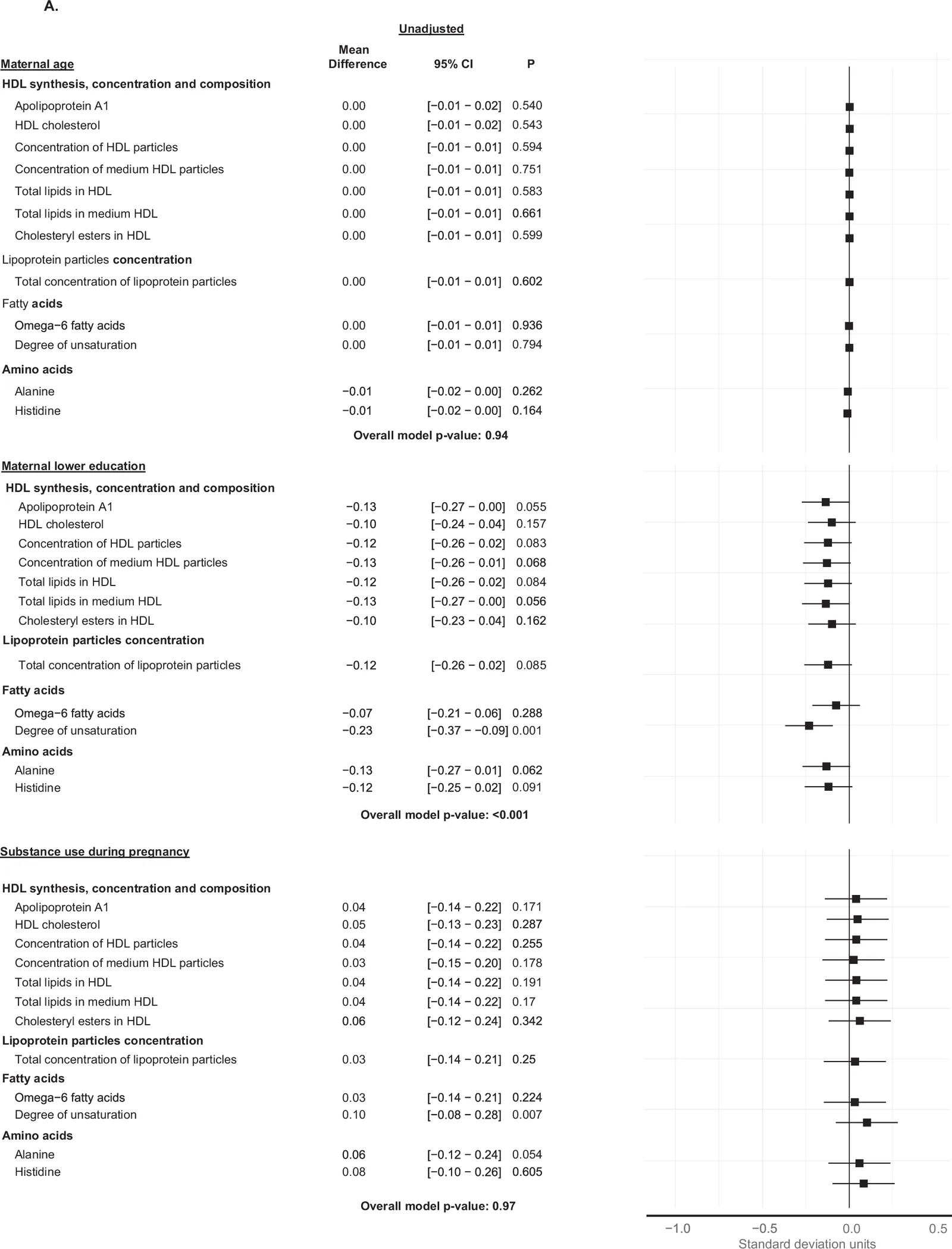

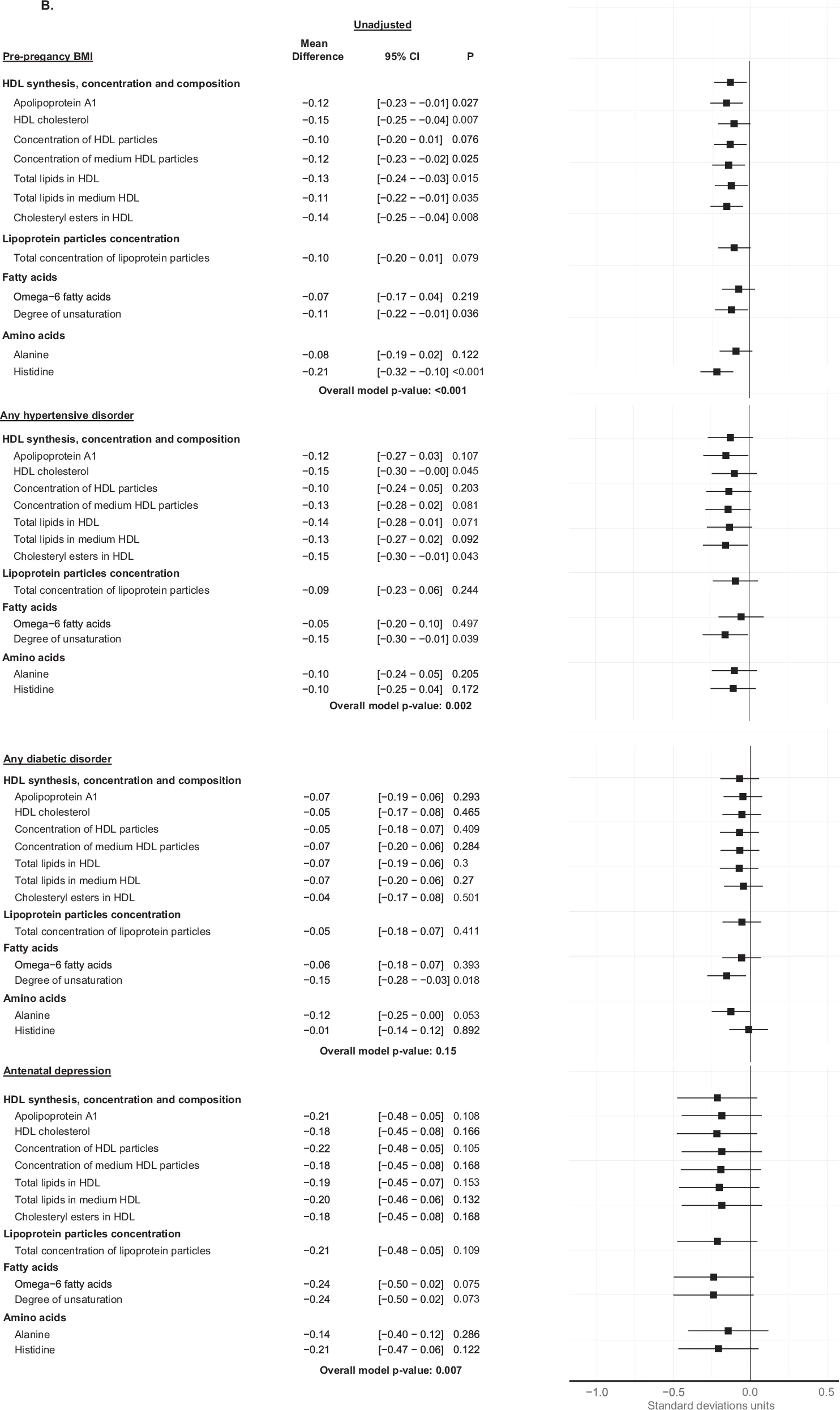

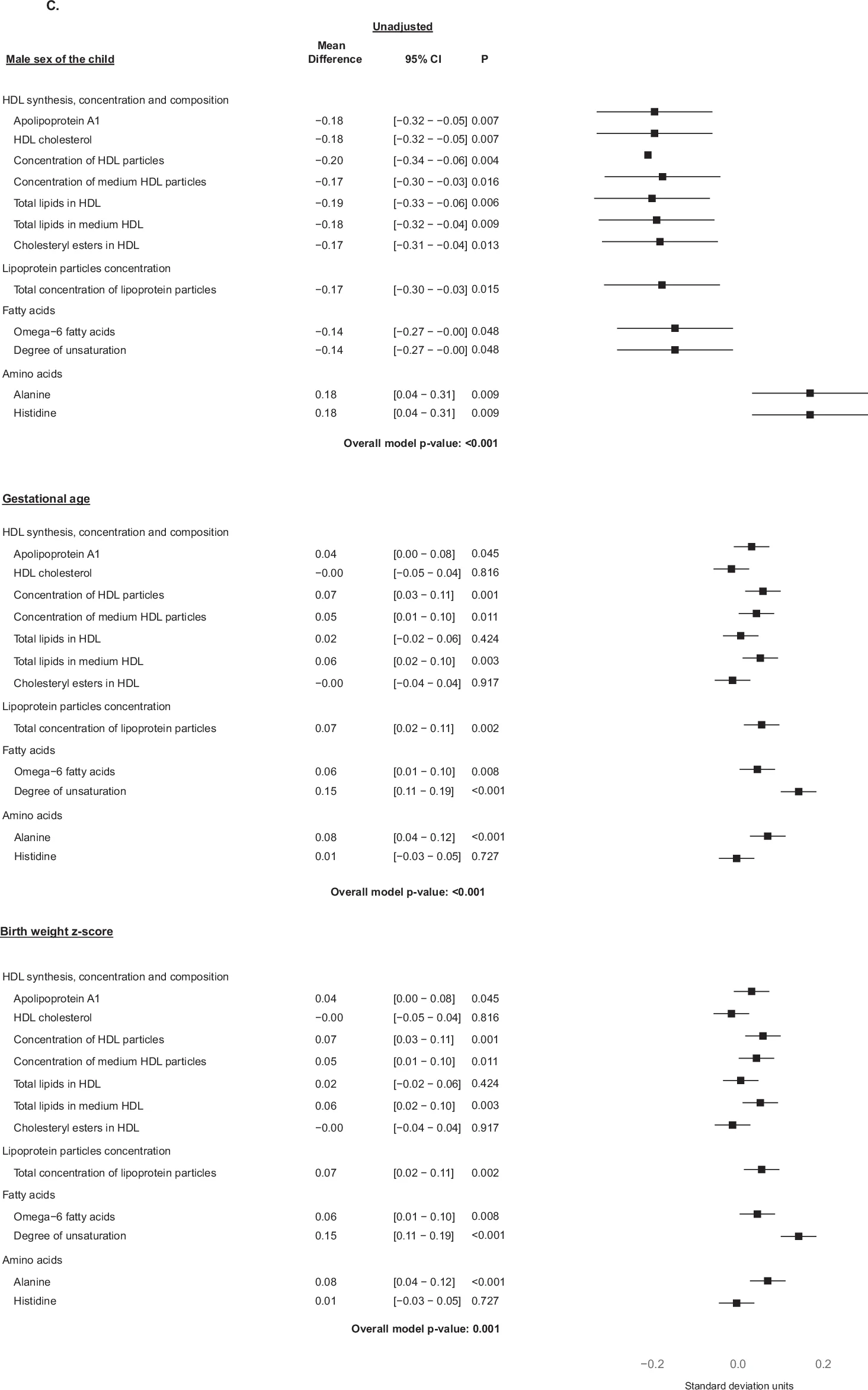

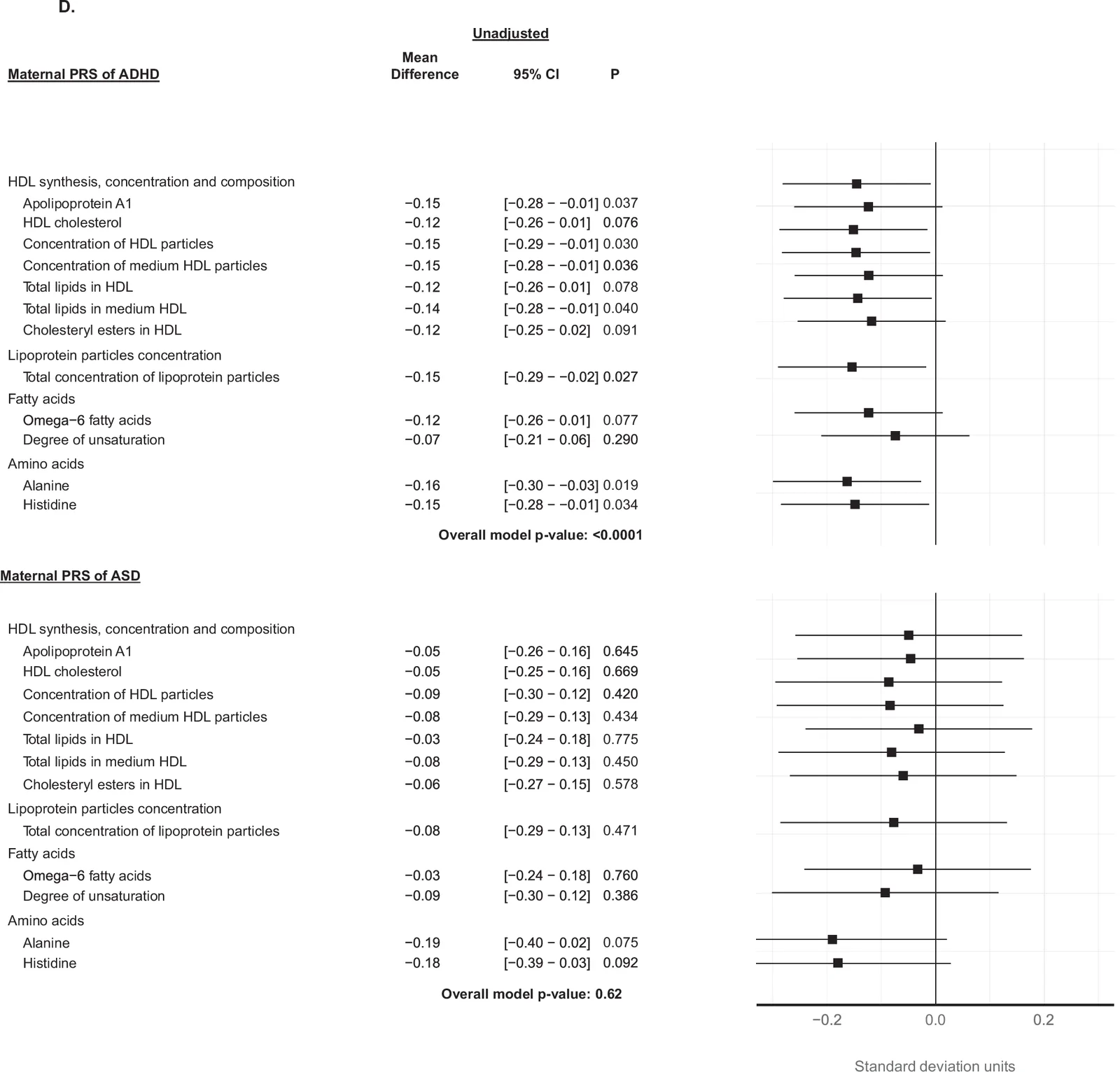
Unadjusted associations of the 12 cord blood metabolic measures with sociodemographic risk factors. Maternal age at delivery (years), education (secondary or lower vs. tertiary), alcohol use and/or smoking during pregnancy (yes vs. no),] (**A**); prenatal risk factors: pre-pregnancy BMI (10kg/m2), diabetic and hypertensive disorders during pregnancy (yes vs. no), antenatal depression (yes vs. no) (**B**); birth outcomes: child sex (male vs. female), gestational age (weeks) and birth weight z-score (SD) (**C**); and maternal genetic liabilities for ADHD and ASD: PRS for ADHD and PRS for ASD (**D**). The forest plots display unadjusted mean differences in metabolic measures in standard deviation units for categorical risk factors and change in metabolic measures in standard deviation units per one-unit increase in continuous risk factors (boxes) and 95% confidence intervals (error bars).

Figure 3 shows the models testing associations between the 12 metabolic measures and sociodemographic risk factors (Panel A), prenatal risk factors (Panel B), birth outcomes (Panel C), and maternal PRSs for ADHD and ASD (Panel D). Figure 3 shows that the overall pattern of associations of the 12 metabolic measures with lower maternal education, higher pre-pregnancy BMI, hypertensive disorders, antenatal depression, and child male sex, lower gestational age, lower birth weight z-score, and higher PRS of ADHDm was consistent with the pattern observed for any NDDs, except for alanine and histidine which were in the opposite direction among male newborns.

### Performance of the models predicting any NDD

In logistic regression models, the 12 metabolic measures improved model fit compared with a model that included sociodemographic and prenatal risk factors and birth outcomes, as indicated by increases in the AUC and Nagelkirke R² for the amount of variance explained in any NDDs, as well LRT p-value (0.04) (Table 2). Addition of the metabolic measures resulted in modest improvement of the model fit over a model that additionally included maternal PRSs for ADHD and ASD, as shown by increases in the AUC and Nagelkerke R²; however, the LRT p-value was 0.08. (Table 2).

**Table 2:** Performance of the models predicting any neurodevelopmental disorders based on early-life risk factors (N = 858)^a^ and (N = 737)^b^ plus 12 cord blood metabolic measures^c^.

Any neurodevelopmental disorder	AUC (95% CI)	Nagelkirke R^2^	p	Sensitivity	Specificity	PPV	NPV	LRT^d^ p
Early life risk factors^a^	0.71 (0.66, 0.76)	12.2	<0.0001	0.52	0.75	0.25	0.91	
Early life risk factors^a^ plus 12 metabolic measures	0.74 (0.69, 0.79)	16.6	<0.0001	0.60	0.77	0.29	0.92	0.04
Early life risk factors^b^	0.72 (0.67, 0.78)	13.9	<0.0001	0.46	0.79	0.27	0.90	
Early life risk factors^b^ plus 12 metabolic measures	0.75 (0.70, 0.80)	18.3	<0.0001	0.52	0.81	0.32	0.91	0.08

^a^Early-life risk factors included sociodemographic risk factors (maternal age at delivery, education, smoking and alcohol use during pregnancy, child sex), prenatal risk factors (maternal pre-pregnancy body mass index, diabetes and hypertensive disorders during pregnancy, and antenatal depression) and adverse birth outcomes (child gestational age at birth and birth weight z-score).

^b^Early-life risk factors additionally included maternal polygenic liabilities for ADHD and ASD (maternal PRS for ADHD and ASD).

^c^12 metabolic measures included alanine, apolipoprotein A1, HDL cholesterol, cholesteryl esters in HDL, total lipids in HDL, concentration of HDL particles, histidine, total lipids in medium HDL, concentration of medium HDL particles, omega-6 fatty acids, total concentration of lipoprotein particles, degree of unsaturation.

^d^LRT - Likelihood Ratio Test.

## Discussion

We found that lower levels of 12 cord blood metabolic measures were associated with an increased risk of any NDDs in children followed up from birth to age 12.4–16.8 years. These measures included metabolites related to HDL synthesis, concentration, and composition (apolipoprotein A1, HDL cholesterol, HDL particle concentration, concentration of medium HDL particles, total lipids in HDL and medium HDL, and cholesteryl esters in HDL), overall lipoprotein particle concentration, FA (omega-6 fatty acids and degree of unsaturation), and AA (alanine and histidine). In analyses of the most prevalent specific NDDs, lower levels of these metabolic measures were also associated with ADHD, speech and language disorders, ASD and motor development disorders. In analyses of subclinical symptoms, they were associated with scores indicative of neurodevelopmental delay and ASD. These associations were not explained by early-life risk factor of NDDs, including maternal age, education, substance use during pregnancy, pre-pregnancy BMI, diabetic and hypertensive disorders during pregnancy, antenatal depression, child’s sex, gestational age, birth weight z-score, or maternal genetic liability for ADHD or ASD.

Our study also showed that, although early-life risk factors did not explain the associations between the 12 cord blood metabolic measures and NDDs, the overall pattern of associations between early-life risk factors and the 12 cord blood metabolic measures closely mirrored the metabolic profile linked to increased NDD risk. Specifically, maternal lower education, higher pre-pregnancy BMI, hypertensive disorders during pregnancy, antenatal depression, child male sex, lower gestational age, lower birth weight z-score and higher maternal PRS for ADHD were all associated with a cord blood metabolic profile indicative of increased vulnerability to NDDs.

Furthermore, our study showed that the prediction of NDDs was improved in models that included the 12 cord blood metabolic measures in addition to early-life risk factors. Findings from the logistic regression models demonstrated that the 12 cord blood metabolic measures explained a higher proportion of variance (16.6 vs. 12.2%) beyond that accounted for by the early-life risk factors. Although the proportion of variance explained by metabolic measures increased in comparison to a model that in addition to sociodemographic, prenatal and birth-related factors included maternal PRSs for ADHD and ASD (18.3 vs. 13.9%), the LRT p-value did not reach the conventional threshold for statistical significance. This may partly reflect the smaller sample size in the PRS-adjusted analysis, which would reduce statistical power to detect incremental improvement in model fit.

Our findings suggesting that alterations in cord blood lipid metabolism [17, 22] and AA pathways [22–24] may predict neurodevelopmental adversity are supported by previous evidence. While our study is not the first to explore biomarkers in cord blood that may help identify children at risk of developing NDDs later in life, it advances the existing literature in several important ways. We identified a set of metabolites from a much larger panel of neurodevelopmentally relevant metabolic measures than has been used in most prior research and broadened the focus beyond ASD and ADHD to include all NDDs. Additionally, we provided novel insights into the biological mechanisms that may link the early-life risk factors to NDDs. Importantly, our study took a further step by demonstrating that the 12 identified metabolic measures may have clinical relevance, as they increased the variance explained in any NDDs beyond early-life risk factors alone and may serve as early biomarkers for NDDs, potentially enabling earlier diagnosis, intervention, and prevention.

These findings align with our earlier work in a subsample of the same study cohort, where we demonstrated that many of the NDD-associated metabolites in cord blood, including metabolites related to HDL synthesis, concentration and composition, FA and AA, were also lower in pregnant mothers with a higher pre-pregnancy BMI [8, 48]. Additionally, we found that maternal antenatal depression was associated with lower levels of HDL cholesterol, alanine and histidine, along with a higher ratio of saturated FA to total FA in the pregnant mothers [5]. Both BMI- and antenatal depression–related changes in maternal blood were associated with higher risk of mental and behavioral disorders in the children. Together with the present findings, this provides further evidence that prenatal risk factor–related metabolic changes in the mother may reduce the availability of HDL cholesterol, AA and FA for fetal uptake, and thereby alter fetal metabolome in a way that may alter fetal brain development and increase the vulnerability to NDDs in later life. Moreover, a recent study has found that associations between western dietary pattern during pregnancy and neurodevelopmental disorders in childhood and adolescence was mediated by metabolic changes [49], further corroborating this hypothesis.

One possible biological pathway may include steroid hormone biosynthesis, as HDL cholesterol can serve as a cholesterol source for the synthesis of pregnenolone and downstream synthesis of mineralocorticoids, glucocorticoids and sex steroids. Therefore, cholesterol must be adequately supplied to mitochondria for normal steroid hormone production [50] to ensure appropriate hormonal milieu that support optimal brain function [51, 52].

The cord blood metabolites identified as potential NDD biomarkers in our study have been linked to mental health problems in adult populations, with the direction of these associations aligning with our findings. Seven of the 12 identified metabolites in cord blood were related to HDL cholesterol synthesis, concentration and composition, and evidence suggests that low HDL cholesterol is associated with aggression, suicide attempts, and major depression in adult populations [5, 53–55]. Additionally, higher levels of alanine and histidine have been suggested to confer protective effects against age-related cognitive and neurological disorders [56, 57] while higher levels of unsaturated FAs have been associated with reduced risks of ADHD, ASD, bipolar disorder, cognitive impairment, and schizophrenia [58]. Our findings highlight that metabolic changes associated with neurodevelopmental problems may be shaped by early-life exposures and can be detected as early as birth. Further research is needed to determine whether the NDD-associated metabolic profile established during gestation and evident at birth persists across the lifespan.

Our results identify candidate metabolic measures that, pending further validation, may in the future help inform prevention and early intervention strategies. Although the findings are based on predictive associations and do not establish causality, they highlight metabolic markers that could potentially be modifiable through targeted interventions. Existing literature suggests that several of the metabolites associated with increased NDD risk in our study may be influenced by dietary or nutritional factors, though the clinical utility of such modifications remains a subject for future investigation. Previous evidence, although not entirely conclusive, suggests that altering the levels of lipids, AA and FA associated with the risk of NDDs can at least alleviate the symptoms. For example, a clinical trial found that dietary supplementation with unsaturated FA alleviated ADHD symptoms in 8–12 year-old children [59]. However, previous clinical trials have largely focused on altering metabolic measures within a single metabolic pathway, our results provide a rationale for investigating whether a broader approach involving multiple metabolic pathways might be a relevant consideration for future intervention frameworks.

One of the main strengths of our study is the combination of methodological approaches that allowed us to identify cord blood metabolites with the strongest ability to predict NDDs and to assess the contribution of each identified metabolic measure while accounting for their highly correlated nature. Additionally, our methodological approach allowed us to rule out the possibility that the associations between cord blood metabolites and NDDs were confounded by early-life risk factors linked both to cord blood metabolites and NDDs [60, 61]. The metabolomic platform used in this study has been used in many epidemiological studies, and many of the metabolic measures have been validated against clinical chemistry methods [62], which facilitates replication. Other study strengths include large sample size, prospective study design, well-characterized sample, and data on the child outcomes extracted from nationwide registers with nearly null follow-up data attrition.

A limitation of our study is that majority of the women were recruited based on their clinical risk factor status for pre-eclampsia and intrauterine growth restriction resulting in overrepresentation of prenatal environmental adversities in our sample. A further limitation is that the targeted metabolomic panel used in our study, although encompassing multiple metabolic pathways, does not capture the full complexity of the cord blood metabolome. Jointly tuning the elastic-net mixing parameter (tested from 0.1 to 0.9) and the regularization parameter by selecting the combination that minimized cross-validated prediction error does not constitute a fully nested cross-validation framework and therefore may introduce some degree of optimistic bias in performance estimation. To mitigate this concern, we implemented repeated 10-fold cross-validation and additionally assessed the robustness of the selected predictors across folds. Another consideration is that confirmatory BLMM analyses were conducted in the same cohort used for elastic net selection. This may introduce some selection bias in effect estimates and associated p-values; therefore, the results should be interpreted as hypothesis-generating and require replication. Although we were able to use PRSs for ASD and ADHD as proxies, the lack of reliable parental clinical NDD diagnosis data remains a further limitation. Finally, the study was conducted in a high-resource Nordic setting, which limits generalizability.

## Conclusion

Our study identified 12 cord blood metabolic measures that predicted the risk of any NDDs in children followed-up from birth to 12.4–16.8 years of age. These metabolic measures were associated with early-life risk factors for NDDs and improved NDD prediction compared to models including sociodemographic and prenatal risk factors and birth outcomes; however, the improvement was more modest when maternal PRSs for ADHD and ASD were included in the predictive models. These metabolic measures have the potential to become early biomarkers of NDDs, contributing towards timely diagnosis, treatment, and prevention.

## Supplementary information

Supplement 1

## Data Availability

The data are not publicly available due to confidentiality agreements and Finnish data protection law. Health data containing sensitive participant information cannot be disclosed publicly. Qualified researchers may request data access by contacting the corresponding author, subject to institutional approval and execution of a data use agreement.
