# Supplementary material for: Metabolomic alterations in cord blood improve the prediction of childhood-onset neurodevelopmental disorders
Source: Transl Psychiatry. 2026 Jun 25;16:460. doi: 10.1038/s41398-026-04208-x (PMC13558667; doi:10.1038/s41398-026-04208-x)
Supplement: Supplementary file 1 [file 41398_2026_4208_MOESM1_ESM.docx]

Supplement 1.

| eTable 1. International Classification of Diseases and Related Health Problems. 10^th^ Revision (ICD-10) codes included in the definition of neurodevelopmental disorders and their prevalence in the children followed-up from birth to age 12.4-16.8 years. | |
| --- | --- |
| **Category (ICD-10 diagnostic codes)** | **No. (%)** |
| Any neurodevelopmental disorders (F70-F79. F80- F89. F90. F95. F984. F985. F986. F988) | 120 (12.1%) |
| Intellectual development disorders (F70- F79) | 8 (0.9%) |
| Speech and language disorder (F80) | 43 (5.0%) |
| Scholastic skills disorder (F81) | 32 (3.7%) |
| Motor developmental disorder (F82) | 20 (2.3%) |
| Mixed developmental disorder (F83) | 14 (1.6%) |
| Autism Spectrum Disorder (F84) | 24 (2.8%) |
| Other developmental disorder (F88) | 26 (3.0%) |
| Unspecified developmental disorder (F89) | 10 (1.2%) |
| Attention Deficit Hyperactivity Disorder (F90) | 51 (5.9%) |
| Tic disorder (F95) | 11 (1.3%) |
| Stereotyped movement disorder (F98.4) | 0 (0.0%) |
| Stuttering (F98.5) | 0 (0.0%) |
| Cluttering (F98.6) | 0 (0.0%) |
| Other specified behavioral and emotional disorders  with onset in childhood (F98.8) | 4 (0.5%) |

EFigure1.

Age (years)

Number at risk

Age (years)

Number at risk

Age (years)

Number at risk

Age (years)

Number at risk

Age (years)

Number at risk

Age (years)

Number at risk

| ETable 2. Subclinical symptoms of neurodevelopmental disorders in the children | |
| --- | --- |
|  | **Mean (SD)** |
| **Age 2.0-5.7 years** |  |
| Developmental milestones (ASQ) (N=460) | 268.9 (30.7) |
|  |  |
| **Age 7.0-12.1 years** |  |
| Autism spectrum symptoms (ASSQ) (N=420) | 0.12 (0.19) |
| ADHD symptoms (ADHD rating scale) (N=415) | 10.6 (8.8) |
|  |  |
| ASQ refers to Ages and Stages-3 Questionnaire questionnaires^1,2^. ASSQ refers to Autism Spectrum Screening Questionnaire^3^ and ADHD rating scale to ADHD symptoms rating scale^4^. | |

| ETable 3. Descriptive statistics of cord blood metabolic measures. | | | | | |
| --- | --- | --- | --- | --- | --- |
| **Metabolic measure** | **Unit of measurement** | **Mean** | **SD** | **N of samples below LOD** | **Percent of samples below LOD** |
| **Lipoprotein subclasses** |  |  |  |  |  |
| Concentration of chylomicrons and extremely large VLDL particles | mmol/l | 3.8 × 10⁻⁷ | 3.7 × 10⁻⁷ | 3 | 0.35% |
| Concentration of very large VLDL particles | mmol/l | 6.1 × 10⁻⁷ | 7.1 × 10⁻⁷ | 3 | 0.35% |
| Concentration of large VLDL particles | mmol/l | 2.3 × 10⁻⁶ | 1.9 × 10⁻⁶ | 3 | 0.35% |
| Concentration of medium VLDL particles | mmol/l | 7.5 × 10⁻⁶ | 4.8 × 10⁻⁶ | 3 | 0.35% |
| Concentration of small VLDL particles | mmol/l | 1.4 × 10⁻⁵ | 7.1 × 10⁻⁶ | 3 | 0.35% |
| Concentration of very small VLDL particles | mmol/l | 2.9 × 10⁻⁵ | 7.5 × 10⁻⁶ | 3 | 0.35% |
| Concentration of IDL particles | mmol/l | 1.45 × 10⁻⁴ | 3.0 × 10⁻⁵ | 3 | 0.35% |
| Concentration of large LDL particles | mmol/l | 3.3 × 10⁻⁴ | 6.9 × 10⁻⁵ | 3 | 0.35% |
| Concentration of medium LDL particles | mmol/l | 1.2 × 10⁻⁴ | 3.6 × 10⁻⁵ | 3 | 0.35% |
| Concentration of small LDL particles | mmol/l | 8.5 × 10⁻⁵ | 1.5 × 10⁻⁵ | 3 | 0.35% |
| Concentration of very large HDL particles | mmol/l | 1.9 × 10⁻⁴ | 7.1 × 10⁻⁵ | 3 | 0.35% |
| Concentration of large HDL particles | mmol/l | 8.26 × 10⁻⁴ | 5.1 × 10⁻⁴ | 3 | 0.35% |
| Concentration of medium HDL particles | mmol/l | 1.92 × 10⁻³ | 6.9 × 10⁻⁴ | 3 | 0.35% |
| Concentration of small HDL particles | mmol/l | 5.6 × 10⁻³ | 1.1 × 10⁻³ | 3 | 0.35% |
| **Lipoprotein particle concentrations** |  |  |  |  |  |
| Total concentration of lipoprotein particles | mmol/l | 9.3 × 10⁻³ | 2.1 × 10⁻³ | 3 | 0.35% |
| Concentration of VLDL particles | mmol/l | 5.4 × 10⁻⁵ | 2.1 × 10⁻⁵ | 3 | 0.35% |
| Concentration of LDL particles | mmol/l | 5.4 × 10⁻⁴ | 1.3 × 10⁻⁴ | 3 | 0.35% |
| Concentration of HDL particles | mmol/l | 8.6 × 10⁻³ | 2.0 × 10⁻³ | 3 | 0.35% |
| **Lipoprotein particle sizes** |  |  |  |  |  |
| Average diameter for VLDL particles | nm | 35.84 | 1.15 | 3 | 0.35% |
| Average diameter for LDL particles | nm | 23.85 | 0.06 | 3 | 0.35% |
| Average diameter for HDL particles | nm | 9.63 | 0.23 | 3 | 0.35% |
| **Cholesterol** |  |  |  |  |  |
| Total cholesterol | mmol/l | 1.85 | 0.60 | 2 | 0.23% |
| Total cholesterol minus HDL-C | mmol/l | 1.05 | 0.41 | 3 | 0.35% |
| VLDL cholesterol | mmol/l | 0.22 | 0.10 | 3 | 0.35% |
| Clinical LDL cholesterol | mmol/l | 0.66 | 0.35 | 2 | 0.23% |
| LDL cholesterol | mmol/l | 0.51 | 0.23 | 3 | 0.35% |
| HDL cholesterol | mmol/l | 0.80 | 0.23 | 2 | 0.23% |
| Remnant cholesterol (non-HDL. non-LDL -cholesterol) | mmol/l | 0.54 | 0.19 | 3 | 0.35% |
| **Cholesteryl esters** |  |  |  |  |  |
| Total esterified cholesterol | mmol/l | 1.35 | 0.44 | 3 | 0.35% |
| Cholesteryl esters in VLDL | mmol/l | 0.14 | 0.06 | 3 | 0.35% |
| Cholesteryl esters in LDL | mmol/l | 0.35 | 0.17 | 3 | 0.35% |
| Cholesteryl esters in HDL | mmol/l | 0.63 | 0.18 | 3 | 0.35% |
| **Free cholesterol** |  |  |  |  |  |
| Total free cholesterol | mmol/l | 0.50 | 0.15 | 3 | 0.35% |
| Free cholesterol in VLDL | mmol/l | 0.08 | 0.04 | 3 | 0.35% |
| Free cholesterol in LDL | mmol/l | 0.16 | 0.06 | 3 | 0.35% |
| Free cholesterol in HDL | mmol/l | 0.17 | 0.05 | 3 | 0.35% |
| **Triglycerides** |  |  |  |  |  |
| Total triglycerides | mmol/l | 0.46 | 0.22 | 2 | 0.23% |
| Triglycerides in VLDL | mmol/l | 0.26 | 0.16 | 3 | 0.35% |
| Triglycerides in LDL | mmol/l | 0.07 | 0.03 | 3 | 0.35% |
| Triglycerides in HDL | mmol/l | 0.07 | 0.03 | 3 | 0.35% |
| **Phospholipids** |  |  |  |  |  |
| Total phospholipids in lipoprotein particles | mmol/l | 1.48 | 0.34 | 3 | 0.35% |
| Phospholipids in VLDL | mmol/l | 0.14 | 0.07 | 3 | 0.35% |
| Phospholipids in LDL | mmol/l | 0.23 | 0.07 | 3 | 0.35% |
| Phospholipids in HDL | mmol/l | 0.97 | 0.23 | 3 | 0.35% |
| **Apolipoproteins** |  |  |  |  |  |
| Apolipoprotein B | g/l | 0.38 | 0.08 | 2 | 0.23% |
| Apolipoprotein A1 | g/l | 0.92 | 0.19 | 2 | 0.23% |
| Ratio of apolipoprotein B to apolipoprotein A1 | ratio | 0.42 | 0.08 | 2 | 0.23% |
| **Other lipids** |  |  |  |  |  |
| Phosphoglycerides | mmol/l | 1.08 | 0.31 | 3 | 0.35% |
| Ratio of triglycerides to phosphoglycerides | ratio | 0.43 | 0.17 | 3 | 0.35% |
| Total cholines | mmol/l | 1.30 | 0.33 | 3 | 0.35% |
| Phosphatidylcholines | mmol/l | 0.96 | 0.27 | 3 | 0.35% |
| Sphingomyelins | mmol/l | 0.27 | 0.06 | 3 | 0.35% |
| **Total lipids** |  |  |  |  |  |
| Total lipids in lipoprotein particles | mmol/l | 3.79 | 1.03 | 3 | 0.35% |
| Total lipids in VLDL | mmol/l | 0.62 | 0.32 | 3 | 0.35% |
| Total lipids in LDL | mmol/l | 0.82 | 0.32 | 3 | 0.35% |
| Total lipids in HDL | mmol/l | 1.84 | 0.47 | 3 | 0.35% |
| **Fatty acids** |  |  |  |  |  |
| Total fatty acids | mmol/l | 5.09 | 1.44 | 3 | 0.35% |
| Degree of unsaturation | degree | 1.39 | 0.10 | 3 | 0.35% |
| Omega-3 fatty acids | mmol/l | 0.26 | 0.15 | 4 | 0.47% |
| Omega-6 fatty acids | mmol/l | 2.16 | 0.45 | 4 | 0.47% |
| Polyunsaturated fatty acids | mmol/l | 2.42 | 0.55 | 4 | 0.47% |
| Monounsaturated fatty acids | mmol/l | 0.76 | 0.41 | 4 | 0.47% |
| Saturated fatty acids | mmol/l | 1.91 | 0.55 | 4 | 0.47% |
| Linoleic acid | mmol/l | 0.97 | 0.38 | 4 | 0.47% |
| Docosahexaenoic acid | mmol/l | 0.16 | 0.07 | 4 | 0.47% |
| **Fatty acids ratios** |  |  |  |  |  |
| Ratio of omega-3 fatty acids to total fatty acids | % | 4.79 | 1.93 | 4 | 0.47% |
| Ratio of omega-6 fatty acids to total fatty acids | % | 43.44 | 4.79 | 4 | 0.47% |
| Ratio of polyunsaturated fatty acids to total fatty acids | % | 48.23 | 3.88 | 4 | 0.47% |
| Ratio of monounsaturated fatty acids to total fatty acids | % | 14.28 | 4.11 | 4 | 0.47% |
| Ratio of saturated fatty acids to total fatty acids | % | 37.49 | 1.76 | 4 | 0.47% |
| Ratio of linoleic acid to total fatty acids | % | 18.85 | 3.68 | 4 | 0.47% |
| Ratio of docosahexaenoic acid to total fatty acids | % | 3.16 | 0.85 | 4 | 0.47% |
| Ratio of polyunsaturated fatty acids to monounsaturated fatty acids | ratio | 3.98 | 5.11 | 4 | 0.47% |
| Ratio of omega-6 fatty acids to omega-3 fatty acids | ratio | 11.49 | 8.00 | 4 | 0.47% |
| **Glycolysis related metabolites** |  |  |  |  |  |
| Glucose | mmol/l | 4.21 | 1.37 | 4 | 0.47% |
| Lactate | mmol/l | 5.18 | 2.40 | 3 | 0.35% |
| Pyruvate | mmol/l | 0.14 | 0.12 | 109 | 12.70% |
| Citrate | mmol/l | 0.08 | 0.02 | 2 | 0.23% |
| **Amino acids** |  |  |  |  |  |
| Alanine | mmol/l | 0.36 | 0.11 | 2 | 0.23% |
| Glutamine | mmol/l | 0.57 | 0.14 | 110 | 12.82% |
| Glycine | mmol/l | 0.16 | 0.06 | 3 | 0.35% |
| Histidine | mmol/l | 0.11 | 0.02 | 3 | 0.35% |
| **Branched-chain amino acids** |  |  |  |  |  |
| Total concentration of branched-chain amino acids | mmol/l | 0.39 | 0.09 | 5 | 0.58% |
| Isoleucine | mmol/l | 0.05 | 0.02 | 2 | 0.23% |
| Leucine | mmol/l | 0.11 | 0.03 | 2 | 0.23% |
| Valine | mmol/l | 0.23 | 0.05 | 5 | 0.58% |
| **Aromatic amino acids** |  |  |  |  |  |
| Phenylalanine | mmol/l | 0.07 | 0.01 | 4 | 0.47% |
| Tyrosine | mmol/l | 0.07 | 0.02 | 3 | 0.35% |
| **Ketone bodies** |  |  |  |  |  |
| 3-Hydroxybutyrate | mmol/l | 0.13 | 0.12 | 12 | 1.40% |
| Acetate | mmol/l | 0.05 | 0.02 | 3 | 0.35% |
| Acetoacetate | mmol/l | 0.02 | 0.03 | 3 | 0.35% |
| Acetone | mmol/l | 0.02 | 0.02 | 2 | 0.23% |
| **Fluid balance** |  |  |  |  |  |
| Creatinine | μmol/l | 53.12 | 13.58 | 3 | 0.35% |
| Albumin | g/l | 38.98 | 8.58 | 2 | 0.23% |
| **Inflammation** |  |  |  |  |  |
| Glycoprotein acetyls | mmol/l | 0.43 | 0.10 | 2 | 0.23% |
| Metabolic measures below level of detection (LOD) were replaced with a value of 0.9 multiplied by the minimum value of that measurement^5^. All metabolic measures were log-transformed and standardized to the mean of 0 and standard deviation (SD) of 1. All values above and below 5 standard deviations (SDs) from the mean were considered outliers and recoded as missing values^6^. Missing values were not imputed and were treated as missing in all analyses. | | | | | |

| ETable 4. Raw values of 12 NDDs-related cord blood metabolic measures disorders in the children with neurodevelopmental disorders and in children with no mental or behavioral disorders in a follow-up from birth to 12.4-16.8 years. | | | | |
| --- | --- | --- | --- | --- |
|  |  | **No mental or behavioral disorders** | **Any NDD** |  |
|  | **Unit of measurement** | **Mean (SD)** | **Mean (SD)** | **p** |
| HDL synthesis, concentration and composition | |  |  |  |
| Apolipoprotein A1 | g/l | 0.93 (0.19) | 0.86 (0.15) | 0.0008 |
| HDL cholesterol | mmol/l | 0.81 (0.24) | 0.74 (0.19) | 0.001 |
| Concentration of HDL particles | mmol/l | 8.65 × 10⁻³ (2.06 × 10⁻³) | 7.97 × 10⁻³ (1.67 × 10⁻³) | 0.0006 |
| Concentration of medium HDL particles | mmol/l | 1.96 × 10⁻³ (7.08 × 10⁻⁴) | 1.72 × 10⁻³ (5.68 × 10⁻⁴) | 0.0006 |
| Total lipids in HDL | mmol/l | 1.86 (0.48) | 1.71 (0.38) | 0.001 |
| Total lipids in medium HDL | mmol/l | 0.52 (0.17) | 0.52 (0.14) | 0.0008 |
| Cholesteryl esters in HDL | mmol/l | 0.64 (0.18) | 0.58 (0.14) | 0.0009 |
| Lipoprotein particles concentration |  |  |  |  |
| Total concentration of lipoprotein particles | mmol/l | 9.39 × 10⁻³ (2.17 × 10⁻³) | 8.69 × 10⁻³ (1.76 × 10⁻³) | 0.0008 |
| Fatty acids |  |  |  |  |
| Omega-6 fatty acids | mmol/l | 2.2 (0.46) | 2.0 (0.35) | 0.0009 |
| Degree of unsaturation | degree | 1.39 (1.0) | 1.36 (0.09) | 0.002 |
| Amino acids |  |  |  |  |
| Alanine | mmol/l | 0.37 (0.11) | 0.34 (0.08) | 0.01 |
| Histidine | mmol/l | 0.11 (0.02) | 0.10 (0.02) | 0.01 |

| ETable 5. Early-life risk factors of neurodevelopmental disorders in the children with neurodevelopmental disorders and in children with no mental or behavioral disorders in a follow-up from birth to 12.4-16.8 years in a subsample with available maternal polygenic liability scores for ADHD and ASD | | | | | | | |
| --- | --- | --- | --- | --- | --- | --- | --- |
| **Early-life risk factors** | **No mental or behavioral disorders**  **(N=633)** | **Any NDD**  **(N=104)** | **ADHD**  **(N=44)** | **Speech and language disorders**  **(N=38)** | **Scholastic skills disorders**  **(N=28)** | **ASD**  **(N=20)** | **Motor development**  **disorders**  **(N=19)** |
|  | M (SD) or No. (%) | M (SD) or No. (%) | M (SD) or No. (%) | M (SD) or No. (%) | M (SD) or No. (%) | M (SD) or No. (%) | M (SD) or No. (%) |
| **Sociodemographic risk factors** |  |  |  |  |  |  |  |
| Maternal age, years | 33.2 (5.6) | 32.8 (6.0) | 31.2 (6.3)* | 33.1 (6.0) | 30.5 (6.8 )* | 33.2 (5.7) | 32.9 (6.7) |
| Maternal education, No. (%) |  | *** | * | * | *** |  | * |
| Primary or secondary education | 255 (43.0%) | 60 (59.4%) | 26 (63.4%) | 23 (62.1%) | 21 (80.8%) | 7 (36.8%) | 13 (72.2%) |
| University degree | 352 (57.0%) | 41 (40.6%) | 15 (36.6%) | 14 (37.8%) | 5 (19.2) | 12 (63.2%) | 5 (27.8%) |
| Data not available | 16 (2.5%) | 3 (2.9%) | 3 (6.8%) | 1 (2.6%) | 1 (3.6%) | 1 (5.0%) | 1 (5.0%) |
| Maternal smoking and/or alcohol use during pregnancy, No. (%) |  |  |  |  |  |  |  |
| No | 521 (82.3%) | 83 (79.2%) | 36 (81.8%) | 33 (86.8%) | 25 (89.3%) | 16 (80.0%) | 15 (79.0%) |
| Yes | 112 (17.7%) | 21 (20.2%) | 8 (18.2%) | 5 (13.2%) | 3 (10.7%) | 4 (20.0%) | 4 (21.0%) |
| **Prenatal risk factors** |  |  |  |  |  |  |  |
| Maternal pre-pregnancy body mass index, kg/m^2^ | 27.0 (6.2) | 30.0 (6.5)*** | 31.2 (6.8)*** | 30.0 (6.0)** | 30.5 (7.2)* | 27.7 (4.4) | 31.9 (5.5)*** |
| Maternal diabetes disorders during pregnancy, No. (%) |  | * |  | * |  |  | ** |
| No | 466 (73.6%) | 63 (60.6%) | 26 (59.1%) | 22 (57.9%) | 17 (60.7%) | 14 (70.0%) | 9 (47.4%) |
| Yes | 167 (26.4%) | 41 (39.4%) | 18 (40.9%) | 16 (42.1%) | 11 (39.3%) | 6 (30.0%) | 10 (52.6%) |
| Maternal hypertensive disorders during pregnancy, No. (%) |  |  |  | ** |  |  |  |
| No | 420 (66.4%) | 66 (63.5%) | 26 (59.1%) | 19 (50%) | 19 (76.9%) | 13 (65.0%) | 10 (52.6%) |
| Yes | 213 (33.6%) | 38 (36.5%) | 18 (40.9%) | 19 (50%) | 9 (32.1%) | 7 (35.0%) | 9 (47.4%) |
| Maternal antenatal depression, No. (%) |  | *** | *** | *** | ** | *** |  |
| No | 598 (94.5%) | 88 (84.6%) | 36 (81.8%) | 30 (79.0%) | 23 (82.1%) | 15 (75.0%) | 16 (84.2%) |
| Yes | 35 (5.5%) | 16 (15.4%) | 8 (18.2%) | 8 (21.0%) | 5 (17.9%) | 5 (25.0%) | 3 (15.8%) |
| **Polygenic liabilities for ADHD and ASD** |  |  |  |  |  |  |  |
| Polygenic risk score for ADHD of the mother, SD | -0.05 (1.00) | 0.21 (0.96)* | 0.28 (0.91)* | 0.32 (1.03)* | 0.26 (0.94) | 0.31 (0.82) | 0.25 (1.06) |
| Polygenic risk score for ASD of the mother, SD | -0.02 (0.99) | 0.00 (1.02) | 0.14 (1.11) | -0.05 (0.98) | -0.20 (1.18) | 0.35 (1.10) | -0.09 (1.27) |
| **Birth outcomes** |  |  |  |  |  |  |  |
| Child sex, No. (%) |  | *** | *** | *** | ** | *** | ** |
| Boy | 317 (50.1%) | 77 (74.0%) | 37 (84.1%) | 32 (84.2%) | 22 (78.6%) | 17 (85.0%) | 15 (79.0%) |
| Girl | 316 (49.9%) | 27 (26.0%) | 7 (15.9%) | 6 (15.8%) | 6 (21.4%) | 3 (15.0%) | 4 (21.0%) |
| Child gestational age at birth, weeks | 39.8 (1.7) | 39.7 (1.8) | 39.7 (1.6) | 39.7 (2.0) | 39.8 (1.6) | 39.7 (2.3) | 39.2 (2.4) |
| Birth weight z-score, standard deviation units | 0.00 (1.04) | 0.25 (1.1)* | 0.23 (1.08) | 0.28 (1.17) | 0.41 (0.92)* | 0.20 (1.20) | 0.25 (1.26) |
| There is no missing data unless indicated otherwise  Comparison of characteristics between children with any NDD or specific NDDs and children without mental or behavioral disorder diagnoses: *p<0.05 **p<0.01 ***p<0.001 | | | | | | | |

| **ETable 6. Mean differences in the 12 cord blood metabolic measures between children with any neurodevelopmental disorders and those with no mental disorders. The models are adjusted for early-life risk factors for neurodevelopmental disorders and maternal PRS for ADHD and ASD.** | | | | |
| --- | --- | --- | --- | --- |
| Metabolic measure | Estimate | 95% CI | | P |
| HDL synthesis, concentration and composition |  |  |  |  |
| Apolipoprotein A1 | -0.34 | -0.56 | -0.13 | 0.0016 |
| HDL cholesterol | -0.31 | -0.52 | -0.10 | 0.0046 |
| Concentration of HDL particles | -0.36 | -0.57 | -0.14 | 0.0011 |
| Concentration of medium HDL particles | -0.33 | -0.55 | -0.12 | 0.0024 |
| Total lipids in HDL | -0.32 | -0.53 | -0.11 | 0.0034 |
| Total lipids in medium HDL | -0.34 | -0.55 | -0.12 | 0.0019 |
| Cholesteryl esters in HDL | -0.32 | -0.53 | -0.10 | 0.0038 |
| Lipoprotein particles concentration |  |  |  |  |
| Total concentration of lipoprotein particles | -0.36 | -0.57 | -0.14 | 0.0011 |
| Fatty acids |  |  |  |  |
| Omega-6 fatty acids | -0.38 | -0.59 | -0.16 | 0.0005 |
| Degree of unsaturation | -0.30 | -0.51 | -0.08 | 0.0066 |
| Amino acids |  |  |  |  |
| Alanine | -0.29 | -0.51 | -0.08 | 0.0070 |
| Histidine | -0.32 | -0.53 | -0.10 | 0.0037 |

**Efigure 2.**

1.0000

0.4261

0.3071

0.3140

0.3605

0.4898

0.5035

0.4380

0.4225

0.5280

0.4996

0.4338

0.4261

1.0000

0.9562

0.9504

0.9794

0.9717

0.5179

0.9825

0.9767

0.9219

0.9681

0.6503

0.3071

0.9562

1.0000

0.9952

0.9843

0.8966

0.4820

0.9334

0.9390

0.8356

0.8873

0.6013

0.3140

0.9504

0.9952

1.0000

0.9845

0.9028

0.4866

0.9382

0.9443

0.8356

0.8921

0.6113

0.3605

0.9794

0.9843

0.9845

1.0000

0.9393

0.4860

0.9690

0.9689

0.8905

0.9337

0.6192

0.4898

0.9717

0.8966

0.9028

0.9393

1.0000

0.5375

0.9873

0.9804

0.9502

0.9984

0.6858

0.5035

0.5179

0.4820

0.4866

0.4860

0.5375

1.0000

0.5224

0.5150

0.5252

0.5345

0.4839

0.4380

0.9825

0.9334

0.9382

0.9690

0.9873

0.5224

1.0000

0.9967

0.9234

0.9808

0.6740

0.4225

0.9767

0.9390

0.9443

0.9689

0.9804

0.5150

0.9967

1.0000

0.9137

0.9732

0.6757

0.5280

0.9219

0.8356

0.8356

0.8905

0.9502

0.5252

0.9234

0.9137

1.0000

0.9605

0.6736

0.4996

0.9681

0.8873

0.8921

0.9337

0.9984

0.5345

0.9808

0.9732

0.9605

1.0000

0.6794

0.4338

0.6503

0.6013

0.6113

0.6192

0.6858

0.4839

0.6740

0.6757

0.6736

0.6794

1.0000

Ala

ApoA1

HDLC

HDLCE

HDLL

HDLP

His

MHDLL

MHDLP

Omega6

TotalP

Unsaturation

Unsaturation

TotalP

Omega6

MHDLP

MHDLL

His

HDLP

HDLL

HDLCE

HDLC

ApoA1

Ala

0.4

0.6

0.8

Pearson Correlation

**Abbreviations:**

Ala - Alanine

ApoA1 - Apolipoprotein A1

HDLC - HDL cholesterol

HDLCE - Cholesteryl esters in HDL

HDLL - Total lipids in HDL

HDLP - Concentration of HDL particles

His -Histidine

MHDLL - Total lipids in medium HDL

MHDLP - Concentration of medium HDL particles

Omega6 - Omega-6 fatty acids

TotalP - Total concentration of lipoprotein particles

Unsaturation - Degree of unsaturation

Apolipoprotein A1

HDL cholesterol

Concentration of HDL particles

Concentration of medium HDL particles

Total lipids in HDL

Total lipids in medium HDL

Cholesteryl esters in HDL

Total concentration of lipoprotein particles

Omega−6 fatty acids

Degree of unsaturation

Alanine

Histidine

HDL synthesis, concentration and composition

Lipoprotein particles concentration

Fatty acids

Amino acids

0.10

0.11

0.09

0.11

0.11

0.10

0.12

0.09

0.08

0.05

0.01

0.11

[0.01 − 0.19]

[0.02 − 0.20]

[0.00 − 0.18]

[0.02 − 0.20]

[0.01 − 0.20]

[0.01 − 0.19]

[0.02 − 0.21]

[−0.00 − 0.18]

[−0.02 − 0.17]

[−0.04 − 0.15]

[−0.08 − 0.10]

[0.01 − 0.20]

0.034

0.017

0.049

0.016

0.024

0.028

0.013

0.056

0.101

0.241

0.866

0.025

Apolipoprotein A1

HDL cholesterol

Concentration of HDL particles

Concentration of medium HDL particles

Total lipids in HDL

Total lipids in medium HDL

Cholesteryl esters in HDL

Total concentration of lipoprotein particles

Omega−6 fatty acids

Degree of unsaturation

Alanine

Histidine

HDL synthesis, concentration and composition

Lipoprotein particles concentration

Fatty acids

Amino acids

−0.14

−0.16

−0.13

−0.15

−0.15

−0.14

−0.17

−0.12

−0.10

−0.20

−0.07

−0.11

[−0.25 − −0.02]

[−0.28 − −0.04]

[−0.25 − −0.01]

[−0.27 − −0.03]

[−0.26 − −0.03]

[−0.26 − −0.02]

[−0.29 − −0.05]

[−0.24 − −0.00]

[−0.22 − 0.02]

[−0.32 − −0.08]

[−0.19 − 0.05]

[−0.23 − 0.01]

0.024

0.007

0.034

0.013

0.016

0.019

0.005

0.048

0.089

0.001

0.244

0.074

**ASD symptoms**

Apolipoprotein A1

HDL cholesterol

Concentration of HDL particles

Concentration of medium HDL particles

Total lipids in HDL

Total lipids in medium HDL

Cholesteryl esters in HDL

Total concentration of lipoprotein particles

Omega−6 fatty acids

Degree of unsaturation

Alanine

Histidine

HDL synthesis, concentration and composition

Lipoprotein particles concentration

Fatty acids

Amino acids

−0.04

−0.06

−0.03

−0.05

−0.05

−0.05

−0.06

−0.03

−0.02

−0.06

0.03

−0.06

[−0.14 − 0.07]

[−0.16 − 0.04]

[−0.14 − 0.07]

[−0.16 − 0.05]

[−0.15 − 0.06]

[−0.15 − 0.06]

[−0.17 − 0.04]

[−0.13 − 0.08]

[−0.12 − 0.09]

[−0.16 − 0.05]

[−0.08 − 0.13]

[−0.17 − 0.04]

0.481

0.253

0.526

0.321

0.375

0.373

0.227

0.594

0.773

0.289

0.597

0.224

**ADHD symptoms**

−1.0	−0.5	0.0	0.5

Standard deviations

**Overall model p-value: 0.04**

**Overall model p-value: 0.008**

**Overall model p-value: 0.66**

**References**

1.	Squires J, Bricker D, Potter L. Revision of a parent-completed development screening tool: Ages and Stages Questionnaires. *J Pediatr Psychol* 1997; **22**(3): 313-28.

2.	Squires J, Bricker DD, Twombly E. Ages & stages questionnaires: Paul H. Brookes Baltimore; 2009.

3.	Posserud MB, Lundervold AJ, Gillberg CJJocp, psychiatry. Autistic features in a total population of 7–9‐year‐old children assessed by the ASSQ (Autism Spectrum Screening Questionnaire). 2006; **47**(2): 167-75.

4.	Zhang S, Faries DE, Vowles M, Michelson D. ADHD rating scale IV: psychometric properties from a multinational study as clinician-administered instrument. 2005; **14**(4): 186-201.

5.	Seah JYH, Hong Y, Cichońska A, et al. Circulating Metabolic Biomarkers Are Consistently Associated With Type 2 Diabetes Risk in Asian and European Populations. *The Journal of Clinical Endocrinology & Metabolism* 2022; **107**(7): e2751-e61.

6.	Kivelä J, Sormunen-Harju H, Girchenko PV, et al. Longitudinal metabolic profiling of maternal obesity, gestational diabetes and hypertensive pregnancy disorders. *J Clin Endocrinol Metab* 2021.

**EFigures legends**

EFigure 1. Cumulative incidence of neurodevelopmental disorders diagnoses

EFigure 2. Pearson correlations between the 12 NDD-associated metabolic measures

EFigure 3. Unadjusted associations between the 12 cord blood metabolic measures and child subclinical symptoms of neurodevelopmental disorders. The forest plots display change in metabolic measures in standard deviation units per standard deviation unit change in subclinical symptoms) (boxes) and 95% Confidence Intervals (error bars).
